# The preoperative flexion tear gap affects postoperative meniscus stability after pullout repair for medial meniscus posterior root tear

**DOI:** 10.1186/s43019-025-00264-7

**Published:** 2025-04-03

**Authors:** Masanori Tamura, Takayuki Furumatsu, Takahiro Kitayama, Yusuke Yokoyama, Yuki Okazaki, Koki Kawada, Toshifumi Ozaki

**Affiliations:** 1 Department of Orthopaedic Surgery, Okayama University Graduate School of Medicine, Dentistry, and Pharmaceutical Sciences, Okayama, 700-8558 Japan; 2 Department of Orthopaedic Surgery, Japanese Red Cross Okayama Hospital, 2-1-1 Aoe, Okayama, Kitaku 700-8607 Japan; 3 Department of Radiology, Okayama University Hospital, 2-5-1 Shikatacho, Okayama, Kitaku 700-8558 Japan

**Keywords:** Medial meniscus, Posterior root tear, Distance, Pullout repair, Second-look arthroscopy

## Abstract

**Background:**

We investigated whether the preoperative flexion tear gap (FTG) observed in open magnetic resonance imaging (MRI) affects meniscus stability after medial meniscus (MM) posterior root (MMPR) repairs. Furthermore, time-correlated MRI findings from MMPR tear occurrence were evaluated.

**Methods:**

This retrospective observational study included 54 patients (mean age, 64.6 years; 13 males and 41 females) who underwent pullout repair for radial degenerative MMPR tear. Meniscus stability (scored 0–4 points) was assessed using a semi-quantitative arthroscopic scoring system during second-look arthroscopy 1 year postoperatively. The FTG was evaluated on preoperative axial MRI at 90° knee flexion. Other MRI measurements included MM extrusion (MME) at 10° knee flexion, MM posterior extrusion (MMPE) at 90° knee flexion, and MM posteromedial extrusion (MMpmE) at 90° knee flexion preoperatively and 1 year postoperatively. The correlation between the arthroscopic stability score and MRI findings was investigated. A receiver-operating characteristic curve was calculated to predict a good meniscus healing score (3–4 points). The correlation between the FTG and patient demographics, including time from injury to MRI, was analyzed.

**Results:**

At 1 year postoperatively, MME increased by 1.1 mm, while MMpmE and MMPE decreased by 0.4 mm and 1.0 mm, respectively. The meniscus stability score was negatively correlated with the preoperative FTG (r = -0.61, p < 0.01). The time from injury to MRI was significantly correlated with the preoperative FTG. The receiver-operating characteristic curve identified an FTG cut-off value of 8.7 mm for predicting good postoperative stability, with sensitivity and specificity of 67% and 85%, respectively.

**Conclusions:**

FTG evaluated with open MRI at 90° knee flexion was associated with time from injury and affected meniscus stability following pullout repair. MMPR tears should be treated in the early phase to increase meniscus healing stability.

**Supplementary Information:**

The online version contains supplementary material available at 10.1186/s43019-025-00264-7.

## Background

Medial meniscal posterior root (MMPR) tears (MMPRTs) have been increasingly recognized as a significant contributor to the progression of knee osteoarthritis (OA) owing to meniscal extrusion and subchondral insufficiency fractures [1–5]. While transtibial pullout repair is widely used for degenerative MMPRT and is more effective in slowing OA progression compared with conservative treatment and partial meniscectomy, concerns remain regarding its ability to restore the meniscus’s hoop function and prevent the progression of postoperative medial meniscus extrusion (MME) [6–16]. Therefore, surgical indications and methods are still under discussion. Achieving postoperative meniscus stability is critical, because it influences outcomes, such as medial joint space narrowing and cartilage wear [17–19].

There is no consensus on assessing meniscus hoop tension in a clinical setting. MME, as observed on magnetic resonance imaging (MRI) in the extended knee position, might be an indirect indicator of meniscus hoop function, although conflicting results have been reported regarding MME changes after pullout repair [5, 6]. Ultrasound has been proposed to assess dynamic meniscal movements in standing or knee flexion in patients with MMPRT [9, 20, 21]. However, it cannot directly evaluate the degenerative progression of the tear site [22]. Recent studies have reported that open MRI in the knee-flexed position is useful for detecting the pathology and diagnosing early-stage MMPRT [23, 24]. However, its potential for predicting postoperative meniscus stability has not yet been fully explored. Furthermore, the relationship between preoperative open MRI findings, including the tear gap size during knee flexion, and the time from MMPRT occurrence is unclear.

Therefore, this study aimed to investigate whether preoperative MRI findings in knee flexion can predict meniscus stability after pullout repair of MMPRT. As a secondary objective, the relation between the time from injury to MRI and MRI findings, particularly the tear gap in the knee flexion position, was evaluated. We hypothesized that a large tear gap during knee flexion could deteriorate postoperative meniscus stability after healing and that tear gap size would correlate with the time from injury to MRI.

## Methods

### Patients

Our hospital’s institutional review board approved this study (approval number: 1857), and all patients provided written informed consent.

This retrospective observational study included 54 patients (mean age, 64.6 years; 13 males and 41 females) who underwent pullout repair for radial degenerative MMPRT. The inclusion criteria were patients who underwent transtibial pullout repair for radial degenerative MMPRTs (LaPrade type 1 or 2 tears) between April 2018 and April 2022 and underwent pre- and postoperative radiographic assessment, including open MRI. The indication for transtibial pullout repair was persistent knee pain, femorotibial angle ≤ 180°, radiographic Kellgren–Lawrence (K–L) grade 0–2 without subchondral insufficiency fractures on MRI, and mild cartilage lesions (Outerbridge grade 0–2). The exclusion criteria for this study were oblique MMPRTs (LaPrade type 4 tear) and < 2 years of follow-up. We reviewed open MRI data collected pre- and 1 year postoperatively, second-look arthroscopy data collected 1 year after the primary surgery, and annual radiography. All patients accepted the importance of evaluating meniscal healing using second-look arthroscopy with the simultaneous removal of the metal implant and annual radiographic follow-up for OA progression. The time of injury was defined as the time at which the patient experienced a painful popping sensation [25].

### Surgical techniques

**Fig. 1:**
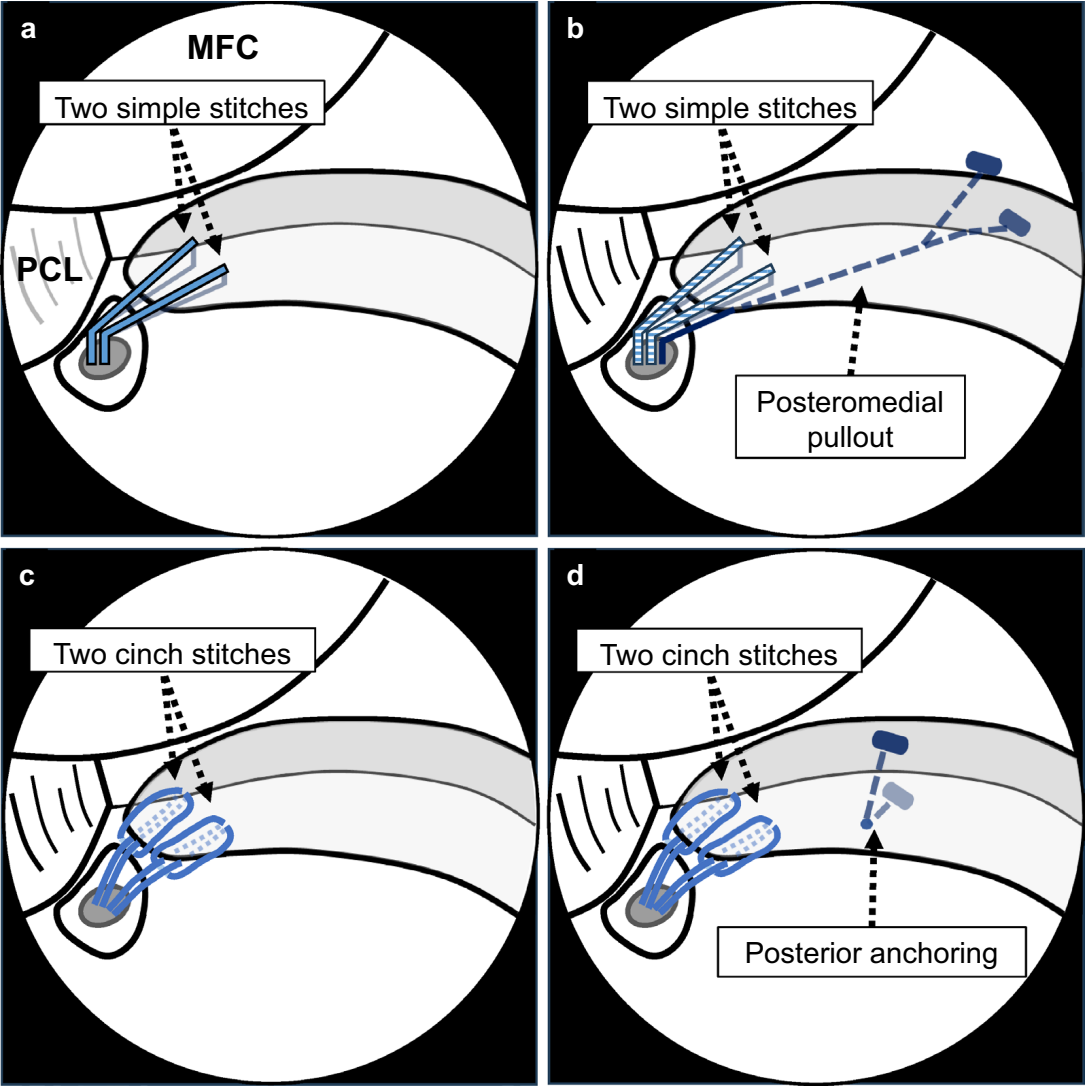
Illustration of four suture configurations. **a** Two simple stitches. **b** Two simple stitches with an additional posteromedial pullout. **c** Two cinch stitches. **d** Two cinch stitches with an additional posterior anchoring. *MFC* medial femoral condyle, *PCL* posterior cruciate ligament

The same experienced orthopedic surgeon performed all surgeries. Four different suture configurations were used as follows: two simple stitches using no. 2 polyethylene sutures between April 2018 and March 2019; two simple stitches with an additional posteromedial pullout technique between April 2019 and June 2020 [26], two cinch stitches using no. 2 polyethylene sutures between July 2020 and December 2021, and two cinch stitches with an additional posterior anchoring technique between January 2021 and April 2021 [27] (Fig. 1). A tibial tunnel was created using dedicated devices, targeting the anatomical footprint of the meniscus root. The pullout sutures were fixed on the tibia using a bioabsorbable interference screw and tied under a metal anchor screw. Patients were initially kept non-weight-bearing in a knee immobilizer for 1–2 weeks postoperatively. Range of motion exercises were initiated at 30°, and flexion was gradually increased (+ 30°/week) to 120°. Partial weight-bearing of < 20 kg was initiated 1–2 weeks postoperatively, with weekly increases of 20 kg until full weight-bearing according to the patient’s weight was achieved. The patients were advised to avoid knee hyperflexion in weight-bearing situations, such as deep squatting, even after meniscal healing.

### Methods of assessment

**Fig. 2:**
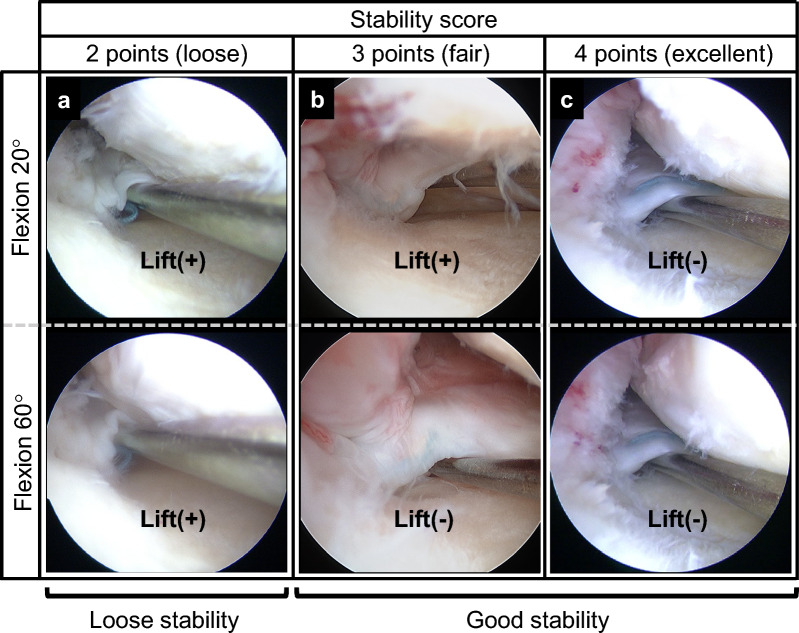
Arthroscopic meniscus stability test of the bridging tissue using probing. **a** Two-point stability; lift on probing at both 20° and 60° knee flexion. **b** Three-point stability; lift on probing at 20° but not at 60° knee flexion. **c** Four-point stability; no lift on probing at either 20° or 60° knee flexion

During second-look arthroscopy, meniscal healing was evaluated using a scoring system on the basis of the following three subscales: stability of bridging tissues, anteroposterior width, and synovial coverage [28]. Stability was carefully evaluated using probing and scored on a 0–4 point scale, with 0 being the worst and 4 being the best. Excellent state (4 points) was defined as continuous meniscus with no lifting on probing during 20° knee flexion. Fair state (3 points) was described as the root that was not raised at knee flexion of 60°, regardless of the degree of lifting during 20° knee flexion. The loose state (2 points) was described as the repaired meniscus with lifting at 60° knee flexion and no anterior drawing at 20° knee flexion. Useless state (1 point) was described as an anterior drawing of the bridging tissue during 20° knee flexion (Fig. 2). A stability score of ≥ 3 was defined as good stability, while a score of ≤ 2 indicated loose stability in this study.

Open MRI evaluation was conducted preoperatively and 1 year postoperatively using an Oasis 1.2 T scanner (Hitachi Medical, Chiba, Japan) with a coil. Patients were kept non-weight-bearing, with the leg placed in 10° and 90° knee-flexed positions. Standard MRI sequences were obtained using a three-dimensional sagittal proton density-weighted sequence with a driven equilibrium pulse and a 90° flip angle. The repetition time/echo time was 500/120 and 600/96 for the 10° and 90° knee-flexed positions, respectively. The slice thickness was 1 mm with no gap. The field of view was 18 cm, and the acquisition matrix size was 224 (phase) × 224 (frequency). Coronal and axial views at knee flexion angles of 10° and 90° were reconstructed for the study using VINCENT software (Fujifilm, Tokyo, Japan). The coronal view was parallel to the femoral condylar axis, and the axial view was parallel to the medial tibial plateau and perpendicular to the sagittal view.

**Fig. 3:**
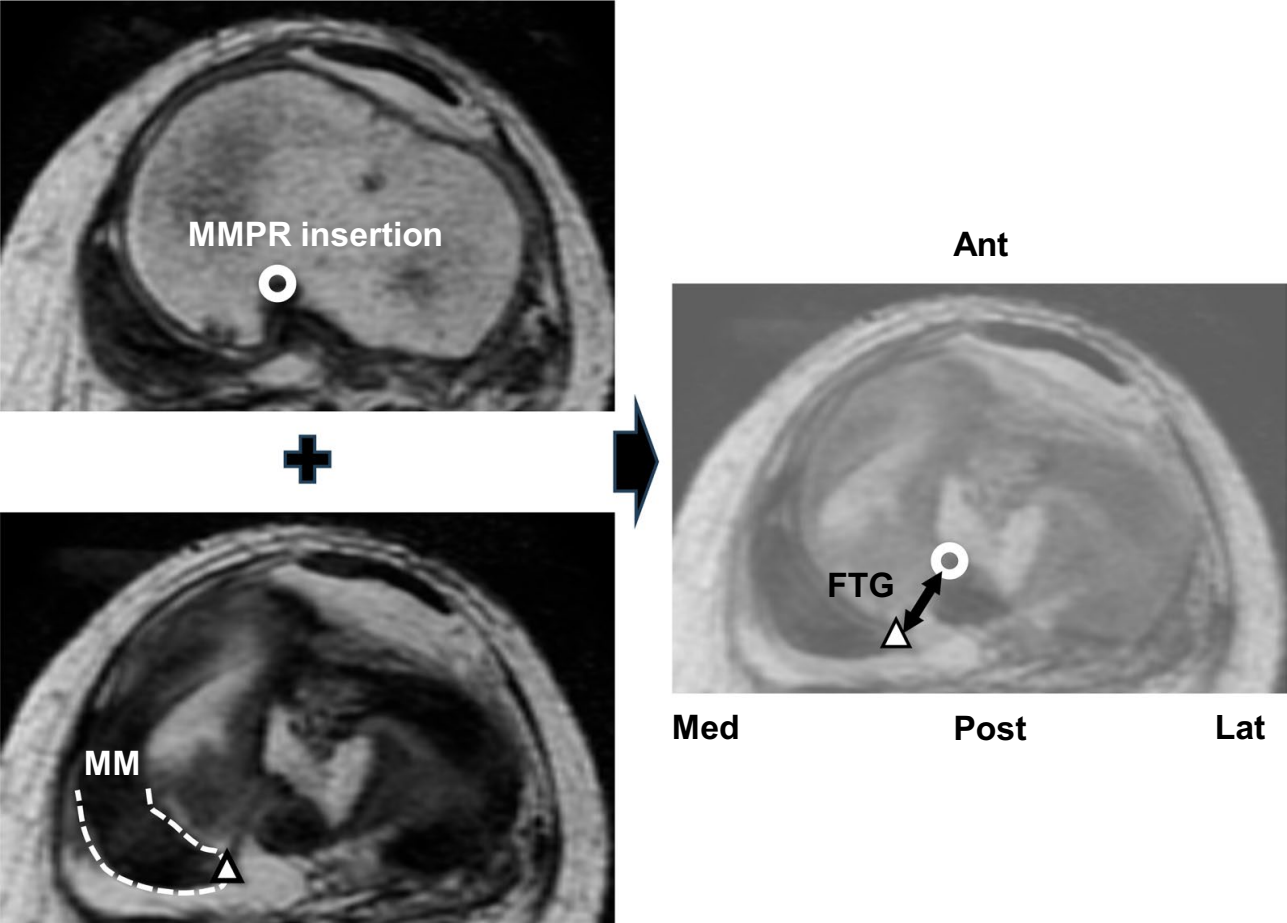
Magnetic resonance imaging measurement of the flexion tear gap (FTG). Image overlay of two axial magnetic resonance images of the knee flexed at 90° shows the FTG. The FTG was measured as the distance between the medial meniscus (MM) posterior root (MMPR) insertion (center of the white circle) and the tip of the tear stump (triangle)

**Fig. 4:**
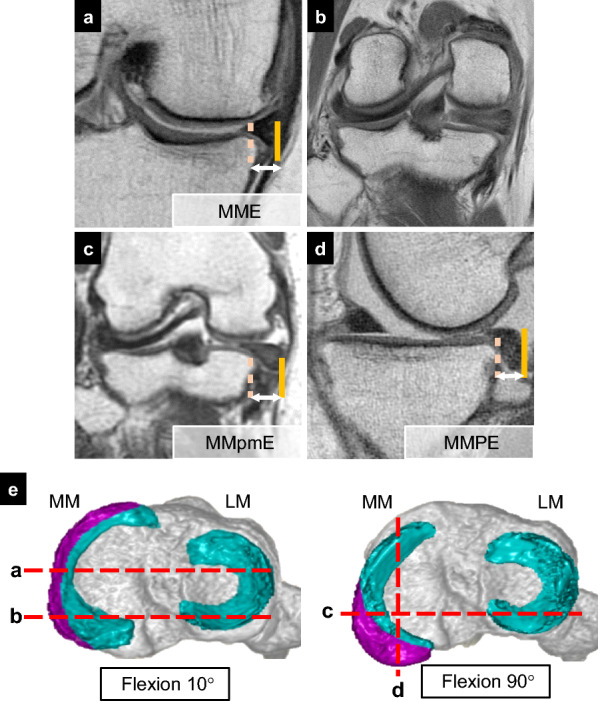
Measurements and reference lines in three-dimensional (3D)-reconstructed MRI of the right knee. MRI images at 10° (**a**, **b**) and 90° (**c**, **d**) knee flexion. A 3D-reconstructed medial meniscus (MM) from above the tibial plateau with the purple area representing the protruding portion from the tibia (**e**). **a**. Medial meniscus extrusion (MME) measured from the tibial edge (dashed line) to the outer edge (line) of the meniscus in the midcoronal plane, excluding osteophytes. **b**. Root tear gap was sometimes not obvious. **c**. Medial meniscus posteromedial extrusion (MMpmE) measured from the tibial edge (dashed line) to the outer edge of the meniscus (line), approximately 4 mm anterior to the posterior edge of the tibial plateau. **d**. Medial meniscus posterior extrusion (MMPE) measured from the tibial edge (dashed line) to the outer edge of the meniscus (line). **e**. Reference lines for** a**–**d**. *MRI* magnetic resonance imaging

The flexion tear gap (FTG) was defined as the distance between the MMPR insertion and lateral tip of the root tear stump in the axial view (Fig. 3). The root insertion and tip of the torn meniscus were confirmed using sagittal and coronal images. The MME, medial meniscus posteromedial extrusion (MMpmE), and MM posterior extrusion (MMPE) were measured from the tibial cartilage edge to the outer edge of the meniscus, excluding the osteophytes (Fig. 4). MME was measured at the midpoint of the anteroposterior length of the MM in the coronal view at 10° knee flexion [29]. MMpmE was measured approximately 4 mm anterior to the posterior edge of the tibial plateau in the coronal view at 90° knee flexion. MMPE was measured at the midpoint of the mediolateral length of the medial tibial plateau in the sagittal view at 90° knee flexion, as previously reported [30]. Subsequently, postoperative changes (ΔMME, ΔMMpmE, and ΔMMPE) were calculated. Intra- and interrater and test–retest reliabilities of the MRI measurements were assessed using the intraclass correlation coefficient (ICC) with a 95% confidence interval. Two blinded examiners evaluated the MRI parameters to assess interrater reliability. Test–retest reliability was assessed by remeasuring MRI parameters 4 weeks after the initial evaluation.

Postoperative radiographic assessment was performed annually using the Rosenberg view [31]. The progression of K–L grade at 2 years postoperatively and MME on MRI at 1 year postoperatively were compared between the two groups according to the good or loose stability at 1-year second-look arthroscopy.

Patient-reported outcome measures were evaluated by research assistants using the Knee Injury and Osteoarthritis Outcome Score (KOOS) before primary surgery and 2 years postoperatively.

### Statistical analysis

Statistical analyses were performed using EZR software (Saitama Medical Center, Jichi Medical University, Saitama, Japan). The Kolmogorov–Smirnov test was used to separate parametric distributions, such as MME, MMpmE, MMPE, patient characteristics, and clinical scores, as well as nonparametric distributions, such as the FTG, duration from injury to MRI or surgery, and second-look arthroscopic scores. Pre- and postoperative MME, MMpmE, MMPE, and clinical scores were compared using paired *t*-tests. Correlations among MRI measurements (FTG, MME, MMpmE, MMPE, and ΔMME), patient characteristics (duration from injury to MRI, age, sex, K–L grade, and femorotibial angle), and stability scores at second-look arthroscopy were evaluated using Spearman’s rank correlation coefficient. Furthermore, the FTG cut-off for predicting postoperative good meniscus stability was calculated using receiver operating characteristic analysis.

Differences in radiographic and clinical outcomes between groups categorized according to good (3 or 4 points) or lax stability (0–2 points) were assessed using the Mann–Whitney *U* test.

As an additional analysis, the surgical techniques in relation to patients’ demographics and MRI findings were compared using the Steel–Dwass test.

FTG measurements were repeated after 4 weeks to assess intraobserver reliability. The interobserver reproducibility and intraobserver repeatability of FTG measurements were satisfactory, with mean ICC values of 0.91 and 0.88, respectively. A post hoc analysis using G*Power (Heinrich-Heine-Universität, Düsseldorf, Germany) was performed to assess the actual power of Spearman’s rank correlation coefficient to evaluate the relationship between stability and FTG. An excellent statistical power of 99.9% was achieved, assuming an effect size of 0.61, α error of 0.05, and sample size of 15.

## Results

Of the 58 patients who underwent surgery, four with oblique MMPRTs were excluded; finally, 54 patients were evaluated. None of the patients underwent further surgery or arthroplasty. Patient demographics and clinical characteristics are presented in Table 1. The time of injury was clear in 45 (76%) patients. The obtained intraoperative stability scores (0, 1, 2, 3, and 4) at 1-year follow-up were 1, 0, 27, 19, and 7, respectively.

**Table 1:** Patient demographics and clinical characteristics and meniscus healing status

Number of patients	54
Sex, male/female	13/41
Age, years (range)	64.6 ± 8.3 (57–71)
Height, m (range)	1.57 ± 0.1 (1.52–1.63)
Weight, kg (range)	62.4 ± 10.3 (54.0–70.0)
Body mass index, kg/m^2^ (range)	25.3 ± 3.3 (22.7–27.0)
Femorotibial angle, ° (range)	177.5 ± 2.3 (176–179)
Preoperative Kellgren–Lawrence grade (0:1:2)	0:25:29
Painful popping episode (%)	76%
Duration from injury to MRI, day (range)	66.1 ± 59.1 (20–105)
Duration from injury to operation, day (range)	80.6 ± 61.2 (36–125)
LaPrade’s classification (1/2/3/4/5)	3/51/0/0/0
Surgical technique (TSS/TSS + PM/TCS/TCS + PA)	12/18/11/13
Arthroscopic meniscus healing score (0/1/2/3/4/5/6/7/8/9/10)	0/0/1/0/1/2/6/17/18/7/2
Width (0/2/4)	0/3/51
Stability (0/1/2/3/4)	1/0/27/19/7
Synovial coverage (0/1/2)	11/36/7

Values are presented as the mean ± standard deviation or number. Range data are presented as first-third quartiles

*TSS* two simple stitches, *PM* posteromedial pullout, *TCS* two cinch stitches, *PA* posterior anchoring, *MRI* magnetic resonance imaging

At 1 year postoperatively, MME had significantly progressed by approximately 1.1 mm, although MMpmE and MMPE decreased by 0.4 mm and 1.0 mm, respectively (Table 2).

**Table 2:** Preoperative and postoperative MRI measurements

	Preoperative	Postoperative	Δ	*p* value
MME, mm	**3.4 ± 0.8**	**4.5 ± 1.2**	1.1 ± 1.0	< 0.01*
MMpmE, mm	**7.3 ± 2.1**	**6.8 ± 2.0**	−0.4 ± 1.6	0.05*
MMPE, mm	**5.4 ± 1.2**	**4.4 ± 1.3**	−1.0 ± 1.3	< 0.01*
FTG, mm	9.8 ± 2.8	N/A	N/A	N/A

Values are presented as the mean ± standard deviation

*MME* medial meniscus extrusion, *MMpmE* medial meniscus posteromedial extrusion, *MMPE* medial meniscus posterior extrusion, *FTG* flexion tear gap, *N/A* not applicable

^*^Values in bold indicate statistical significance (*p* < 0.05)

**Fig. 5:**
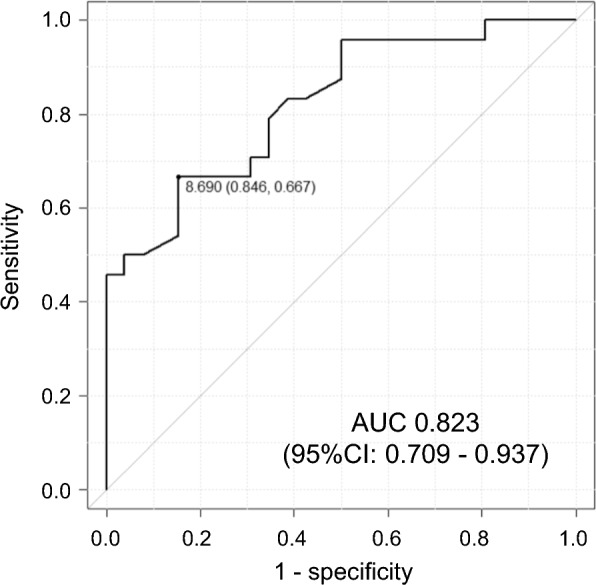
Receiver-operating characteristic curve of the flexion tear gap (FTG) predicting good stability (3, 4 points) The cut-off value of the FTG for good stability was 8.7 mm, with a sensitivity and specificity of 66.7% and 84.6%, respectively. *AUC* area under the curve, *CI* confidence interval

The correlation coefficients between intraoperative meniscus stability score and patient characteristics or radiographic findings are summarized in Table 3. The stability score was negatively correlated with preoperative FTG (*r* = −0.61, *p* < 0.01) and postoperative MME progression (**Δ**MME) (*r* = −0.38, *p* < 0.01). The receiver-operating characteristic curve of the FTG as a predictor of good stability (3 or 4 points) revealed an FTG cut-off of 8.7 mm (sensitivity, 66.7%; specificity, 84.6%) (Fig. 5).

**Table 3:** Correlation coefficients between intraoperative meniscus stability score and patient characteristics or radiographic findings

	Stability (point)	
	Spearman’s coefficient	*p* value
Patient characteristics		
Age	−0.19	N.S
Body mass index	0.09	N.S
Sex	0.25	N.S
Surgical technique [TSS/TSS + PM/TCS/TCS + PA]	−0.07	N.S
Preoperative radiographic findings		
Kellgren–Lawrence grade	−0.05	N.S
Femorotibial angle	0.12	N.S
MME	0.14	N.S
MMpmE	−0.14	N.S
MMPE	0.01	N.S
** FTG**	**−0.61**	< 0.01*
Postoperative radiographic change		
** 1 year ΔMME**	**−0.38**	0.01*

*MME* medial meniscus extrusion, *MMpmE* medial meniscus posteromedial extrusion, *MMPE* medial meniscus posterior extrusion, *FTG* flexion tear gap, Δ = (postoperative − preoperative), *N.S.* not significant

^*^Values in bold indicate statistical significance (*p* < 0.05)

**Fig. 6:**
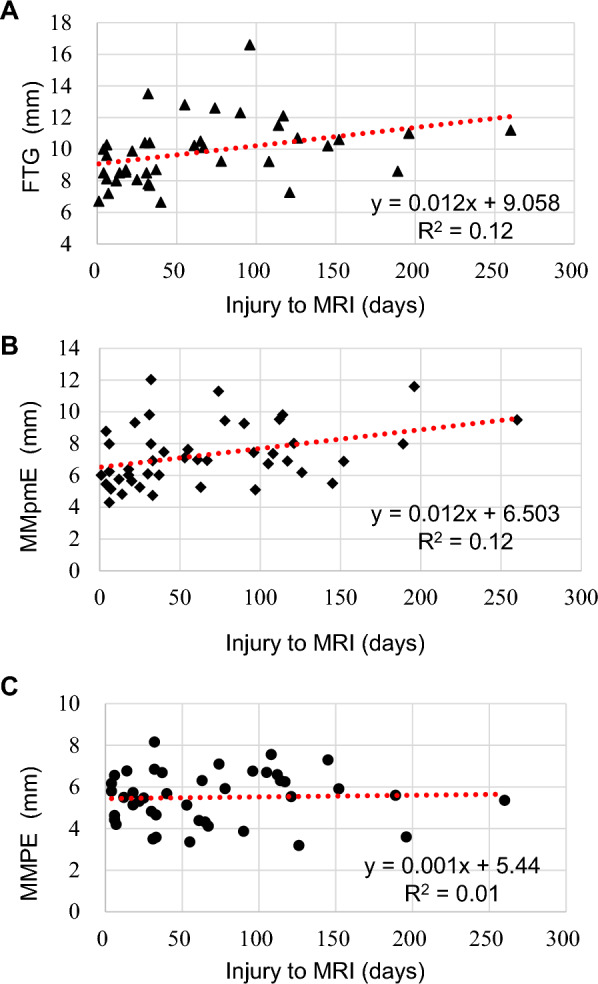
Regression lines of the time-dependent changes in preoperative magnetic resonance imaging (MRI) findings **a**. Moderate correlation between the flexion tear gap (FTG) and time from injury to MRI (R^2^ = 0.12). **b**. Moderate correlation between medial meniscus posteromedial extrusion (MMpmE) and time from injury to MRI (R.^2^ = 0.12) **c**. No correlation between medial meniscus posterior extrusion (MMPE) and time from injury to MRI (R²=0.01)

**Fig. 7:**
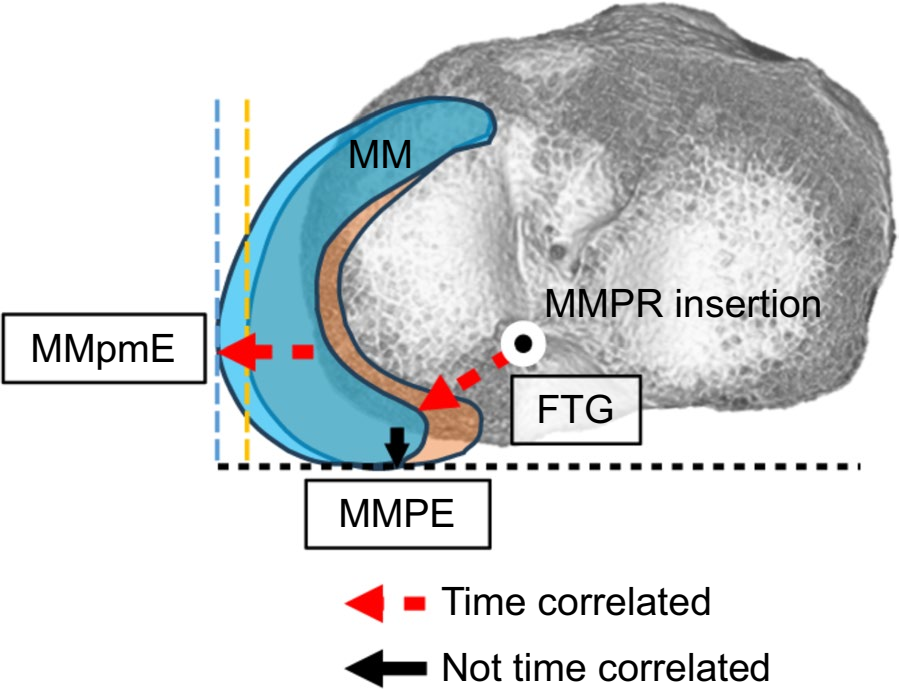
Illustration of time-correlated changes in preoperative magnetic resonance imaging findings with the knee flexed at 90° above the tibial plateau. *MM* medial meniscus, *MMpmE* medial meniscus posteromedial extrusion, *MMPE* medial meniscus posterior extrusion, *FTG* flexion tear gap

In the correlation analysis, FTG significantly correlated with the time from injury to MRI (Table 4). The regression lines for the relationship between time from injury to MRI and preoperative MRI findings (FTG, MMpmE, and MMPE) are shown in Fig. 6, and the time-correlated meniscal movement is illustrated in Fig. 7.

**Table 4:** Correlations of preoperative MRI measurements and patient characteristics

	**FTG**		**MMpmE**		**MMPE**	
	Spearman’s coefficient	*p* value	Spearman’s coefficient	*p* value	Spearman’s coefficient	*p* value
Duration from injury to MRI	**0.40**	< 0.01*	**0.36**	0.01*	0.13	N.S
Age	−0.05	N.S	−0.23	N.S	0.19	N.S
Kellgren–Lawrence grade	0.24	N.S	0.13	N.S	0.26	N.S

*FTG* flexion tear gap, *MMpmE* medial meniscus posteromedial extrusion, *MMPE* medial meniscus posterior extrusion, *MRI* magnetic resonance imaging, *N.S.* not significant

^*^Values in bold indicate statistical significance (*p* < 0.05)

An additional comparison of radiographic findings between the two groups on the basis of meniscus stability revealed that in both groups, MME had significantly progressed at 1 year postoperatively (1.4 mm in the loose stability group versus 0.8 mm in the good stability group) (*p* < 0.05) (Table 5). The rate of K–L grade progression (≥ 1) at 2 years postoperatively was significantly higher in the loose stability group (57% versus 26%).

**Table 5:** Comparison of radiographic assessment between groups classified according to healing stability

	Loose stability (0–2 points, *n* = 28)	Good stability (3 or 4 points, *n* = 26)	*p* value
Preoperative K–L grade (1/2/3/4)	10/18/0/0	10/16/0/0	N.S
2 year postoperative K–L grade (1/2/3/4)	1/18/8/1	6/17/3/0	N.S
K–L grade progression ≧ 1 at 2 years postoperatively (%)	**16 (57%)**	**7 (26%)**	0.03*
K–L grade progression ≧2 at 2 years postoperatively (%)	2 (7%)	0 (0%)	N.S
Preoperative MME, mm	3.3 ± 0.9	3.5 ± 0.8	N.S
1 year postoperative MME, mm	4.8 ± 1.3**	4.3 ± 1.0**	N.S
1 year ΔMME, mm	**1.4 ± 1.1**	**0.8 ± 0.8**	0.02*
Preoperative FTG, mm	**11.2 ± 2.7**	**8.3 ± 2.0**	< 0.01*
Surgical technique (TSS/TSS + PM/TCS/TCS + PA)	6/8/6/8	6/10/5/5	N.S

Values are presented as the mean ± standard deviation or number (%)

*K–L* Kellgren–Lawrance, *MME* medial meniscus extrusion, *FTG* flexion tear gap, *TSS* two simple stitches, *PM* posteromedial pullout, *TCS* two cinch stitches, *PA* posterior anchoring

^*^Values in bold indicate statistical significance (*p* < 0.05)

^**^p < 0.05 (versus preoperative)

Furthermore, an additional comparison of clinical scores between the two groups based on meniscus stability revealed that postoperative KOOS pain score was better in good meniscus stability groups, although all clinical scores significantly improved at 2 years postoperatively in both groups (Table 6).

**Table 6:** Comparison of clinical scores between groups classified according to healing stability

		Loose stability (0–2 points, *n* = 28)	Good stability (3 or 4 points, *n* = 26)	*p* value
KOOS-pain	Preoperative	57.6 ± 15.3	65.8 ± 16.7	N.S
	2 years postoperative	**86.0 ± 12.4**	**92.8 ± 9.3**	0.04*
	*p* value	< 0.01*	< 0.01*	
KOOS-symptoms	Preoperative	61.7 ± 18.8	62.7 ± 18.6	N.S
	2 years postoperative	81.8 ± 14.1	88.5 ± 10.1	N.S
	*p* value	< 0.01*	< 0.01*	
KOOS-ADL	Preoperative	64.8 ± 16.1	66.9 ± 17.6	N.S
	2 years postoperative	85.7 ± 12.7	91.3 ± 8.2	N.S
	*p* value	< 0.01*	< 0.01*	
KOOS-sports/rec	Preoperative	23.1 ± 22.9	25.0 ± 30.4	N.S
	2 years postoperative	51.3 ± 30.0	59.4 ± 29.2	N.S
	*p* value	< 0.01*	< 0.01*	
KOOS-QOL	Preoperative	31.6 ± 20.1	30.4 ± 25.8	N.S
	2 years postoperative	64.8 ± 23.9	68.4 ± 18.9	N.S
	*p* value	< 0.01*	< 0.01*	

Values are presented as the mean ± standard deviation or number (%)

*KOOS* knee injury and osteoarthritis outcome score, *ADL* activities of daily living, *Sport/Rec* sport and recreation, *QOL* quality of life

^*^Values in bold indicate statistical significance (*p* < 0.05)

## Discussion

This study presents two important findings. First, meniscus stability observed during second-look arthroscopy correlated with preoperative FTG. Second, preoperative FTG correlated with the time from injury to MRI. These findings suggest that the preoperative meniscus’ functional position, confirmed via open MRI, affects postoperative meniscus stability after pullout repair and highlights the need for early surgical repair to restore meniscus hoop tension. Furthermore, the good stability group showed significantly less K–L grade progression on radiographic assessment using the Rosenberg view and better clinical scores at 2 years postoperatively.

In this study, a large preoperative FTG was suggested to be a potential factor leading to postoperative loose healing stability. The primary reason for this could be the increased stress on the sutures postoperatively due to the large meniscal movement during knee flexion [32]. Although the initial meniscus fixation tension can be controlled and not too tight initial tension was recommended in a previous report [33], meniscus lax healing and partial suture cutouts, estimated at 40–50%, may be unavoidable after simple MMPR repair in some cases [33, 34]. In total, four different suture techniques for MMPR repair were utilized in this study, which may have affected the initial stability of the repaired meniscus; however, the choice of suture technique did not affect the meniscus healing stability at second-look arthroscopy. Although we aimed to improve meniscus healing by adding simple augmentation (posteromedial pullout or posterior anchoring) using an all-inside device, no clear advantages were observed at 1 year postoperatively (Additional file 1). Recent biomechanical studies have shown that all-inside devices have a lower failure load than two simple stitches [35, 36]. Additionally, postoperative breakage of the posteromedial pullout suture was reportedly up to 75% at second-look arthroscopy 1 year after the primary surgery, suggesting that simple additional augmentation has little effect on meniscus healing.

The novelty of this study lies in identifying the correlation between FTG and MMpmE with the time from injury. Time-dependent MME progression on knee extension MRI after MMPRT onset (0.02 mm/day) has been previously reported [4]. Although exact values cannot be determined without multiple MRI scans from the same patient, a possibility exists that the gap increases by approximately 1.2 mm over 100 days post-injury. Factors contributing to this deterioration may include cartilage wear; progression of local degeneration, such as increased calcification and fibrocartilage formation [22]; altered biomechanical joint contact mechanics owing to impaired meniscus function as a secondary stabilizer [37–39]; and increased stress on surrounding structures, such as the meniscotibial ligament [40]. Further investigations are necessary to better understand the time-dependent changes following injury and their potential role in the progression of OA, as well as to determine whether rehabilitation following injury could help prevent disease progression [41].

Early MMPR repair and an anatomical bone tunnel are crucial for MMPR repair [42]. Moon et al. reported that treatment performed within 13 weeks of symptom onset helped prevent MME progression [3]. Recent advancements, such as centralization or circumferential fiber augmentation, have shown promise in enhancing meniscus fixation and MME reduction in both animal models and human studies [13, 43–45]. However, not all patients might need such augmentation. This study showed that the tear gap at 90° knee flexion is a preoperative factor affecting stability after simple pullout repair. If MMPRTs are diagnosed immediately after onset with a gap of < 8.7 mm at 90°, simple pullout repair may adequately restore meniscal hoop tension. Conversely, for larger tear gaps, additional augmentation techniques may be considered, although further research is needed to confirm these findings.

Despite these novel findings, this study had some limitations. First, the retrospective nature of this study may have led to a selection bias. Second, bone tunnel position was not evaluated in this study. Third, the small number of patients in each surgical group may limit the validity of comparisons between techniques. Fourth, the follow-up period was short; therefore, studies with long-term results are needed to accurately assess postoperative OA progression in each stability groups.

## Conclusions

FTG at 90° knee flexion was associated with time from injury and affected meniscus stability following pullout repair. MMPR tears should be treated in the early phase to increase meniscus healing stability.

## Supplementary Information

Additional file 1. Comparison of surgical techniques in patient’s demographic and MRI findings.

## Data Availability

The datasets generated and analyzed during the current study are available from the corresponding author upon reasonable request.

## Abbreviations

FTG: Flexion tear gap
ICC: Intraclass correlation coefficient
MME: Medial meniscus extrusion
MMPE: Medial meniscus posterior extrusion
MMpmE: Medial meniscus posteromedial extrusion
MMPR: Medial meniscal posterior root
MRI: Magnetic resonance imaging
