# Supplementary material for: The preoperative flexion tear gap affects postoperative meniscus stability after pullout repair for medial meniscus posterior root tear
Source: Knee Surg Relat Res. 2025 Apr 3;37:16. doi: 10.1186/s43019-025-00264-7 (PMC11969855; doi:10.1186/s43019-025-00264-7)
Supplement: Supplementary file 1 — Additional file 1. Comparison of surgical techniques in patient’s demographic and MRI findings. [file 43019_2025_264_MOESM1_ESM.docx]

**Additional files**

Additional file 1. Comparison of surgical techniques in patient’s demographic and MRI findings

|  | TSS  (N = 12) | TSS+PM  (N = 18) | TCS  (N = 11) | TSS+PA  (N = 13) | P value |
| --- | --- | --- | --- | --- | --- |
| **Demographics** |  |  |  |  |  |
| Age | 62.9 ± 10.6 | 63.5 ± 8.4 | 66.5 ± 8.1 | 65.9 ± 6.0 | N.S ^a^ |
| BMI | 25.0 ± 2.8 | 24.6 ± 3.2 | 25.3 ± 3.0 | 26.7 ± 4.1 | N.S ^a^ |
| Duration from injury to surgery | 70.5 ± 49.0 | 80.3 ± 70.1 | 81.4 ± 44.0 | 90.6 ± 76.1 | N.S ^a^ |
| **Pre-operation** |  |  |  |  |  |
| MME, mm | 3.6 ± 0.6 | 3.5 ± 1.0 | 3.4 ± 0.6 | 3.3 ± 0.9 | N.S ^a^ |
| MMpmE, mm | 6.8 ± 1.4 | 8.3 ± 2.6 | 7.1 ± 1.5 | 6.8 ± 1.9 | N.S ^a^ |
| MMPE, mm | 5.3 ± 1.3 | 5.3 ± 1.3 | 5.9 ± 1.4 | 5.2 ± 1.2 | N.S ^a^ |
| FTG, mm | 9.6 ± 1.9 | 10.1 ± 3.4 | 9.3 ± 3.1 | 9.9 ± 2.6 | N.S ^a^ |
| **Post-operation** |  |  |  |  |  |
| 1Y MME, mm | 4.3 ± 0.9 | 4.8 ± 1.4 | 4.1 ± 1.3 | 4.5 ± 1.3 | N.S ^a^ |
| 1Y MMpmE, mm | 5.6 ± 1.7 | 7.7 ± 1.7 | 6.4 ± 1.6 | 6.9 ± 2.3 | N.S ^a^ |
| 1Y MMPE, mm | 4.9 ± 1.4 | 4.1 ± 1.4 | 4.1 ± 1.3 | 4.4 ± 0.9 | N.S ^a^ |
| **Postoperative changes** |  |  |  |  |  |
| Δ MME, mm | 0.7 ± 0.8 | 1.3 ± 1.0 | 0.9 ± 1.2 | 1.3 ± 1.1 | N.S ^a^ |
| Δ MMpmE, mm | -1.0 ± 1.3 | -0.5 ± 1.6 | -0.6 ± 1.0 | 0.1 ± 2.0 | N.S ^a^ |
| Δ MMPE, mm | -0.4 ± 1.2 | -1.2 ± 1.7 | -1.8 ± 0.7 | -0.8 ± 0.8 | N.S ^a^ |

Values are presented as the mean ± standard deviation.

TSS, two simple stitches; PM, posteromedial pullout; TCS, two-cinch stitches; PA, posterior anchoring; MME, medial meniscus extrusion; MMpmE, medial meniscus posteromedial extrusion; MMPE, medial meniscus posterior extrusion; FTG, flexion tear gap; 1Y, 1 year; Δ= (postoperative - preoperative); BMI, body mass index; MRI, magnetic resonance imaging; N.S., not significant

^a^ Statistical differences between the groups were analysed using the Steel-Dwass test
